# Association of Interleukin-1 Beta and Interleukin-1 Receptor Antagonist Gene Polymorphisms and Plasma Levels with Diabetic Nephropathy

**DOI:** 10.1155/2022/9661823

**Published:** 2022-05-25

**Authors:** Xueling Liao, Yanan Xiao, Ulf Elbelt, Karsten H. Weylandt, Kanghui Li, Jie Deng, Ning Zeng, Chao Xue

**Affiliations:** ^1^Department of Nephrology, The Second Affiliated Hospital of Guangxi Medical University, 530007 Nanning, China; ^2^Department of Nephrology, Affiliated Hospital of Guilin Medical College, 541001 Guilin, China; ^3^Division of Medicine, Department of Gastroenterology, Metabolism and Oncology, University Hospital Ruppin-Brandenburg, Brandenburg Medical School, 16816 Neuruppin, Germany; ^4^Medical Department, Division of Psychosomatic Medicine, Campus Benjamin Franklin, Charité-Universitätsmedizin Berlin, Corporate Member of Freie Universität Berlin and Humboldt-Universitätzu Berlin, 12203 Berlin, Germany; ^5^Faculty of Health Sciences, Joint Faculty of the Brandenburg University of Technology, Brandenburg Medical School and University of Potsdam, 14467 Potsdam, Germany

## Abstract

**Objective:**

We investigated the relationships between interleukin- (IL-) 1*β* and IL-1 receptor antagonist (IL-1Ra) gene polymorphism and plasma levels in patients with diabetic nephropathy (DN).

**Methods:**

The genotype and allele frequency distribution of IL-1*β* and IL-1Ra in 61 patients with DN and 48 healthy controls (HCs) were determined by kompetitive allele-specific PCR (KASP), and the plasma concentrations of IL-1*β* and IL-1Ra in DN patients and HCs were measured by enzyme-linked immunosorbent assays (ELISA).

**Results:**

Significant differences were detected in the distribution of IL-1*β* (−511C/T) genotype and allele frequencies between the DN and HC groups (*P* < 0.05), with the T genotype being more frequent in DN patients than HCs (OR = 2.84, 95% CI: 1.489–5.416). The IL-1*β* (+3953C/T) and IL-1Ra (+8006C/T) genotypes and allele frequencies were not significantly different between the two groups (*P* > 0.05). The plasma IL-1*β* level was significantly higher (*P* < 0.01), while the plasma IL-1Ra concentration was significantly lower in the DN group than the HC group (*P* < 0.05). Furthermore, the plasma IL-1*β* level was significantly different between IL-1*β* (−511C/T) locus variants (*P* < 0.05).

**Conclusion:**

The IL-1*β* (−511C/T) gene polymorphism was significantly associated with DN risk in the population of northern Guangxi, China, and the T allele maybe responsible for genetic susceptibility to DN.

## 1. Introduction

Diabetic nephropathy (DN) is one of the most common and severe microvascular complications of type 2 diabetes mellitus (T2DM), and it may progress to end-stage renal disease (ESRD) [1]. To date, the mechanisms underlying the pathogenesis of DN have remained unclear; however, genetic susceptibility and inflammation have been suggested to be critical factors in the development and progression of DN [2].

The interleukin- (IL-) 1 family of cytokines consists of eleven members: IL-1*α*, IL-1*β*, IL-1Ra, IL-18, IL-33, IL-36Ra, IL-36*α*, IL-36*β*, IL-36*γ*, IL-37, and IL-38, which are encoded at three separate loci: two on chromosomes 11 and 9, with the other nine coding genes clustered on the short arm of human chromosome 2 [3, 4]. The main functions of IL-1*β* are proinflammatory; they include the modulation of helper T-cell 17 (Th17) responses and tissue remodeling in many inflammatory and autoimmune diseases [5]. In humans, IL-1*β* has been identified as an important mediator of the inflammatory response [6]. Patients with renal disease have elevated circulating levels of IL-1*β* [7]. Furthermore, IL-1 receptor antagonist (IL-1Ra) is a cytokine for which the only known action is the competitive inhibition of IL-1 binding and thus modulation of the activity of IL-1*β* [8].

In patients with insulin resistance, IL-1 cytokine family and cytokine receptors promote (IL-1*β*)/inhibit (IL-1Ra) macrophage activation [9]. IL-1*β* and IL-1Ra are important mediators of chronic inflammation and tissue damage in multiple organs [10] and are also closely related to T2DM and its complications [11]. Results in a gerbil model showed that high concentrations of glucose induce IL-1*β* production and secretion from human *β*-cells, leading to the upregulation of Fas receptor, NF-*κ*B activation, *β*-cell apoptosis, and renal dysfunction [12]. Similarly, in a mouse model of DN, IL-1*β* caused renal inflammation and deterioration of renal function [13]. Moreover, IL-1Ra may also contribute to renal inflammation in DN with antagonizing effects [14]. Salti et al. [15] suggested that IL-1Ra treatment can prevent the progression and even reverse the evolution of DN in animal models. With increasing understanding, IL-1 family polymorphisms have been used to explain some inherited traits and susceptibility to diabetic disease [11, 16]. A survey in South Korea showed that the IL1B2 and IL1RN∗2 genotypes of the IL-1 gene cluster were associated with DN in Korean patients with T2DM and additionally that carrying these alleles may increase the risk of renal failure [17].

There have been no previous reports regarding the relations between IL-1 polymorphisms and susceptibility to DN in the population of northern Guangxi, China. Here, we investigated the correlations of IL-1*β* and IL-1Ra with DN in a cohort from northern Guangxi. We used the kompetitive allele-specific PCR (KASP) technique to investigate the relations between IL-1*β* and IL-Ra gene polymorphisms and their plasma levels in DN patients and healthy controls (HCs).

## 2. Materials and Methods

### 2.1. Study Participants

The expression level of IL-1*β* in healthy people was 11.7 pg/mL; 48 HCs were collected in this study. PASS 15.0.5 was used to estimate the sample size required for the DN group. The test level *α* was set as 0.5, and the degree of certainty (1 − *β*) was set as 90%; thus, at least 50 cases were needed to calculate the DN group. This case-control study included 61 patients diagnosed with DN at The Affiliated Hospital of Guilin Medical College (Guangxi, China) between January 2020 and July 2020 (34 males and 27 females). A diagnosis of T2DM was made according to the diagnostic and classification criteria for diabetes established by the World Health Organization (WHO) in 1998 [18]: a fasting blood glucose level (FPG) ≥ 7.0 mmol/L and/or oral glucose tolerance test (OGTT) 2 h postprandial blood glucose level ≥ 11.1 mmol/L, a confirmed diagnosis of T2DM, and currently taking oral hypoglycemic drugs or insulin injection treatment. The inclusion criteria for DN were patient state consistent with the diagnosis of diabetes and one of the following conditions: urinary albumin excretion rate (UAER) > 30 mg/24 h at least twice, urinary protein level > 0.5 g/24 h, and serum creatinine > 167 *μ*mol/L. Patients with other diseases that cause proteinuria and renal insufficiency were excluded. During the same period, 48 healthy volunteers were recruited as controls (23 males and 25 females). Individuals with a previous history of diabetes, obesity, hyperlipidemia, arterial hypertension, coronary heart disease, hepatitis, infections, and other diseases were excluded from the study. Subjects who had also recently taken antibiotics and other drugs were excluded. All subjects were genetically unrelated individuals from northern Guangxi, and there were no statistically significant differences in sex, age, or other demographic characteristics between the groups (*P* > 0.05).

The present study was conducted in accordance with the principles of the Declaration of Helsinki, and the study protocol was approved by the Ethics Committee of the Affiliated Hospital of Guilin Medical College. All subjects agreed to have blood withdrawn and provided signed informed consent.

### 2.2. Blood Collection and Processing

#### 2.2.1. Blood Specimens

Venous blood was extracted from fasted subjects into biochemical anticoagulant tubes. After centrifugation at 1000 × *g* for 10 min at room temperature, the plasma supernatant was collected and stored at −80°C.

#### 2.2.2. DNA Isolation

Peripheral venous blood (2 mL) was used for extraction of genomic DNA using a Whole Blood Genomic DNA Extraction Kit (Beijing Adlai Biotechnology Co., Ltd., Beijing, China). Briefly, 900 *μ*L of 1 × red cell lysate was pipetted into a 1.5 mL centrifuge tube, followed by the addition of 300 *μ*L of anticoagulant, and mixed well. The mixture was centrifuged at 12,000 rpm at 4°C for 20 s to obtain the leukocyte precipitate; we then added 300 *μ*L of cell lysate to the resuspended leukocytes. This was followed by the addition of 100 *μ*L of protein precipitation solution and vortex mixing. The mixture was centrifuged at 13,000 rpm for 5 min, and the supernatant was carefully transferred to a fresh 1.5 mL centrifuge tube. Then, 300 *μ*L of isopropanol was added followed by mixing and centrifugation at 12,000 rpm for 1 min. The supernatant was discarded and 1 mL of 70% ethanol was added, followed by centrifugation at 12,000 rpm for 1 min. The resultant precipitate was then dried. The DNA precipitate was solubilized by mixing with 100 *μ*L of DNA lysis solution and then stored at −20°C.

#### 2.2.3. Genotyping

The primers for KASP were designed using Primer5 software (https://primer5.ut.ee/-) and synthesized by Shanghai Bioengineering Co., Ltd. (Shanghai, China). The single nucleotide polymorphism (SNP) of the IL-1*β* (−511C/T) rs16944, the SNP of the IL-1*β* (+3953C/T) rs1143634, and the SNP of the IL-1Ra (+8006C/T) rs419598 were investigated. Target SNPs were analyzed by real-time PCR with the following primers: (1) rs16944F1: 5′-GAAGGTGACCAAGTTCATGCTTGGGTGCTGTTCTCTGCCTCA-3′; rs16944F2: 5′-GAAGGTCGGAGTCAACGGATTGGGTGCTGTTCTCTGCCTCG-3′; and rs16944R: 5′-AGGCTCCTGCAATTGACAGAGAGCT-3′; (2) rs1143634F1: 5′-GAAGGTGACCAAGTTCATGCTGCCTCGTTATCCCATGTGTCG-3′; rs1143634F2: 5′-GAAGGTCGGAGTCAACGGATTAGCCTCGTTATCCCATGTGTCA-3′; and rs1143634R: 5′-CATGTGCTCCACATTTCAGAACCTATCT-3′; and (3) rs419598F1: 5′-GAAGGTGACCAAGTTCATGCTAGGAACAACCAACTAGTTGCT-3′; rs419598F2: 5′-GAAGGTCGGAGTCAACGGATTGGAACAACCAACTAGTTGCC-3′; and rs419598R: 5′-ATTGACATTTGGTCCTTGCAAGTATC-3′. Automated assays were performed using an Applied Biosystems Quant Studio 6 Real-Time PCR System (Foster City, CA, USA). The KASP genotyping PCR amplification reactions were performed under the following conditions: stage 1, predenaturation at 95°C for 15 min; stage 2, denaturation: 95°C for 15 s, annealing: 55°C–65°C, with a temperature ramp of −0.6 Kelvin/cycle for 60 s, 10 cycles; stage 3, extension: 95°C for 15 s, and 55°C for 60 s, 30 cycles; and stage 4, plate reading, 30°C for 30 s, 1 cycle. PCR was performed using a Hydrocycler High Flux Thermal Cycle System. Finally, fluorescence scanning was performed to detect the products.

#### 2.2.4. Measurement of Plasma IL-1*β* and IL-1Ra Levels

The plasma IL-1*β* and IL-1Ra levels were determined by ELISAs (Thermo Multiskan MK3 automatic enzyme marker, provided by Hangzhou Multisciences Co., Hangzhou, Zhejiang, China). All tests were performed in accordance with the manufacturer's instructions.

### 2.3. Statistical Analysis

Statistical analyses were performed using SPSS software ver. 24.0 (IBM Corp., Armonk, NY, USA). The genotype distributions were examined by Hardy-Weinberg equilibrium (HWE) calculation, and allele frequencies were calculated by direct counting. The measurement data are presented as $\overset{¯}{x}\pm \text{s}$, mean ± standard error (SEM), and the counted data are presented as the rate (%). The differences between groups were examined by the *χ*^2^ test, *t*-test, ANOVA, or nonparametric test. The degree of association was expressed as the odds ratio (OR) and 95% confidence interval (CI). In all analyses, *P* < 0.05 was taken to indicate statistical significance.

## 3. Results

The clinical characteristics of the DN and HC groups are summarized in Table 1. A total of 61 DN patients and 48 HC subjects were recruited into this study. There were no significant differences in terms of sex, age, height, body mass index (BMI), blood pressure, and total cholesterol between the two groups. However, the DN patients had higher HbA1c, blood urea nitrogen, creatinine, and triglyceride levels than the HCs (*P* < 0.05).

### 3.1. Main Characteristics and Genotype Distributions of SNPs (HWE) in the DN and HC Groups

The genotype frequencies of IL-1*β* (−511C/T), IL-1*β* (+3953C/T), and IL-1Ra (+8006C/T) were compared between the DN and HC groups by HWE calculation. The differences between the measured and theoretical values of genotype frequencies were not statistically significant (*P* > 0.05), indicating that the distribution of this cohort was under HWE. The genotype frequencies of the three genes and the SNP genotyping plots are shown in Table 2 and Supplementary Figures 1–3.

### 3.2. Associations of IL-1*β* and IL-1Ra Genotype Distributions and Allele Frequencies in DN Patients

Our results show that the numbers of individuals of TT, TC, and CC genotypes of rs16944 (−511C/T) were 46 (75.4%), 11 (18%), and 4 (6.6%) in the DN group and 22 (45.8%), 19 (39.6%), and 7 (14.6%) in the HC group, respectively. Moreover, the frequencies of the T allele of the IL-1*β* gene rs16944 in the DN and HC groups were 84.4% and 65.6%, respectively. Statistical analysis showed that there were significant differences between the two groups in the distribution of IL-1*β* (−511C/T) genotypes (*χ*^2^ = 10.014, *P* < 0.05) and the allele frequencies of IL-1*β* (−511C/T) (*χ*^2^ = 10.456, *P* < 0.01). In addition, an analysis of the allele frequencies showed a 1.84-fold higher frequency for genotypes carrying the T allele at IL-1*β* (−511C/T) compared to those carrying the C allele (OR = 2.84, 95% CI: 1.489–5.416) (Table 3). However, the genotype distribution and allele frequencies of IL-1*β* (+3953C/T) and IL-1Ra (+8006C/T) were not significantly different between the two groups (Table 3).

### 3.3. Evaluation of Plasma IL-1*β* and IL-1Ra Levels

**Figure 1:**
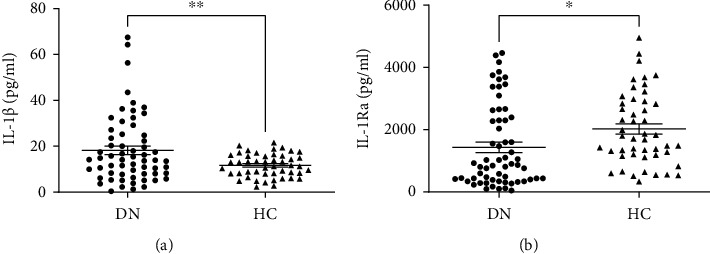
Plasma concentrations of IL-1*β* (a) and IL-1Ra (b) in the DN and HC groups. Data are given as mean ± SEM. Statistical evaluation: *t*-test; ∗ indicates *P* < 0.05; ∗∗ indicates *P* < 0.01.

The plasma IL-1*β* concentration was significantly higher in the DN group than the HC group (18.24 ± 1.88 vs. 11.63 ± 0.72 pg/mL, respectively, *P* < 0.01). In contrast, the plasma IL-1Ra levels were lower in the DN group than in the HC group (1432.20 ± 168 vs. 2026.31 ± 168.6 pg/mL, respectively, *P* < 0.05) (Figure 1).

### 3.4. Evaluation of Plasma IL-1*β* and IL-1Ra Levels with Different Genotypes

**Figure 2:**
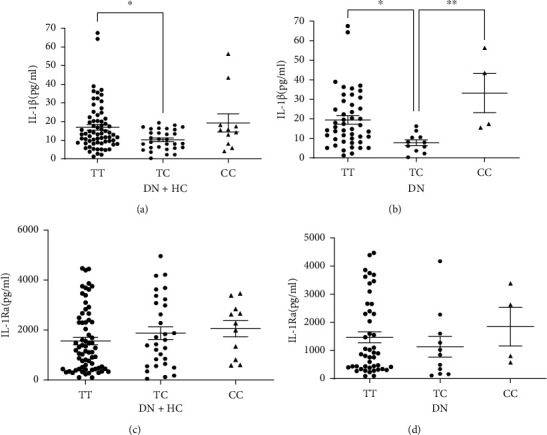
Comparison of plasma IL-1*β* and IL-1Ra levels. (a) IL-1*β* concentration in DN and HCs with different genotypes of IL-1*β* (-511C/T). (b) IL-1*β* concentrations in DN group with different genotypes of IL-1*β* (-511C/T). (c) IL-1Ra concentration in DN and HCs with different genotypes of IL-1*β* (-511C/T). (d) IL-1Ra concentration in DN group with different genotypes of IL-1*β* (-511C/T). Data are given as mean ± SEM. Statistical evaluation: ANOVA analysis; ∗ indicates *P* < 0.05; ∗∗ indicates *P* < 0.01.

We determined the correlations of plasma IL-1*β* concentrations with TT, TC, and CC genotypes in 109 participants to explore the relations between each allele of the IL-1*β* (−511C/T) polymorphism and plasma levels of IL-1*β* and IL-1Ra. The plasma IL-1*β* concentrations were 16.95 ± 1.54 pg/mL for the TT genotype, 10.21 ± 0.98 pg/mL for the TC genotype, and 19.27 ± 4.85 pg/mL for the CC genotype. Thus, the plasma IL-1*β* concentration was significantly higher in subjects with the TT genotype compared with the TC genotype. In addition, the plasma IL-1*β* levels in subjects with TT, TC, and CC genotypes in the DN group were 19.45 ± 2.13, 7.76 ± 1.46, and 33.19 ± 10.02 pg/mL, respectively, indicating that plasma IL-1*β* levels were significantly higher in homozygous compared to heterozygous DN patients. The plasma IL-1Ra levels did not differ significantly between genotypes (Figure 2). Moreover, there were no significant differences in plasma IL-1*β* levels between genotypes for the IL-1*β* (+3953C/T) locus polymorphism and plasma IL-1Ra levels between genotypes for the IL-1Ra (+8006C/T) locus polymorphism (data not shown).

## 4. Discussion

In this study, we examined the polymorphisms of IL-1*β* and IL-1Ra genes in 109 individuals (61 DN patients, 48 HCs). The results show that the IL-1*β* (−511C/T) rs16944 distributions of genotype and allele frequencies were significantly different between the two groups, with the frequency of DN patients with T allele being significantly higher than in the HC group. Furthermore, no significant associations were observed between IL-1*β* (+3953C/T) rs1143634 or IL-1Ra (+8006C/T) rs419598 and DN. To our knowledge, this is the first study to investigate the association of IL-1*β* and IL-1 Ra SNPs with the risk of DN in a population from northern Guangxi.

The anti-inflammatory genes IL-1*β* and IL-1Ra were identified on the long arm of human chromosome 2 [19]. The IL-1*β* gene has two C>T polymorphic sites at promoter −511 and exon+3953, and the IL-1Ra gene has a variable copy number of an 86-bp sequence in intron 2 [20, 21]. Our results are consistent with those of previous studies. An early study demonstrated that the IL-1*β* (−511C/T) polymorphism was associated with DN and that the T allele was more common in patients with DN than in healthy individuals [22]. An additional logistic regression analysis indicated that the IL-1*β* (−511C/T) SNP had an impact on the prevalence of ESRD [23]. A subsequent meta-analysis of 30 published trials concluded that IL-1B −511C/T polymorphism may influence predisposition to DN in both Caucasians and Asians [24].

We further investigated the relationship between IL-1*β* and IL-1Ra gene polymorphisms and plasma IL-1*β* and IL-1Ra concentrations in DN patients and HCs. Our results show that the plasma IL-1*β* levels were significantly higher and plasma IL-1Ra levels were significantly lower in our DN patient cohort than in HCs. These results reinforced earlier work by Niknami et al. [25] showing that DN patients had significantly higher levels of IL-1*β* in comparison to diabetic patients without nephropathy. There is accumulating evidence that the inflammasome and IL-1cytokines are central elements in the pathogenesis of diabetic kidney disease [26, 27]. In particular, IL-1*β* triggers the activation of all types of leukocytes and renal cells, inducing tubulointerstitial fibrosis [28]. Furthermore, in both the total of 109 individuals and in the DN group, the IL-1*β* levels were higher in subjects homozygous for the T allele variant of IL-1*β* (−511C/T) compared with heterozygous individuals. This is consistent with the previous findings by Ioannis et al. [29] showing that a higher plasma IL-1*β* level was associated with the TT genotype. However, the CC allele was found to be associated with a higher IL-1*β* mRNA level through a dominant model [30]. In the present study, IL-1*β* plasma levels were significantly higher for the CC allele than for the TC allele in diabetic patients, but due to the relatively small sample size of patients carrying the CC allele in our diabetic cohort, we were unable to demonstrate an association between the CC allele and the plasma IL-1*β* levels for the TC and TT alleles.

To our knowledge, there have been few studies regarding the relations between IL-1*β* and IL-1 Ra SNPs and DN. Our results strengthen the evidence for a role of the IL-1*β* (−511C/T) polymorphism in DN.

However, this study has several limitations. First, the sample size was limited. Additional studies with larger sample sizes and more detailed studies of the T and C alleles are required to elucidate the impact of the IL-1*β* (−511C/T) gene polymorphism in DN. Second, this study was carried out in a population from northern Guangxi. The genetic association between IL-1*β* (−511C/T) gene polymorphisms and IL-1*β* plasma levels in DN patients may be different in populations of different ethnicities. Third, we cannot exclude the effects of other confounding factors, including nutrition, lifestyle factors, smoking, and treatment factors. Finally, we did not include a group of T2DM patients without DN. Therefore, additional larger studies also including diabetic patients without DN are required to examine whether the changes described above are due to T2DM *per se* or are specific for diabetic patients with DN.

## 5. Conclusions

In conclusion, the present study suggests that the IL-1*β* (−511C/T) polymorphism is significantly correlated with increased DN susceptibility in the population of northern Guangxi, with the T allele as a risk factor, which may contribute to the pathogenesis of DN. Further study is required to validate the clinical significance of these findings.

## Figures and Tables

**Table 1:** Baseline characteristics.

Parameter	DN	HC
Cases	61	48
Gender (M/F)	34/27	23/25
Age (years)	61.39 ± 11.25	57.93 ± 9.84
Height (cm)	165.57 ± 12.61	166.91 ± 28.39
BMI (kg/m^2^)	23.98 ± 5.00	23.88 ± 2.15
SBP (mmHg)	146.15 ± 22.82	128.73 ± 17.15
DBP (mmHg)	87.41 ± 14.51	75.16 ± 10.43
HbA1c (%)	8.83 ± 2.51^∗^	5.56 ± 0.46
UAER (mg/24 h)	2065.71 ± 2269.48	—
BUN (mmol/L)	8.48 ± 6.51^∗^	4.56 ± 1.09
Cr (*μ*mol/L)	177.13 ± 215.45^∗^	70.81 ± 17.11
TG (mmol/L)	2.36 ± 2.11^∗^	1.69 ± 0.86
TC (mmol/L)	4.32 ± 1.36	5.10 ± 1.00

BMI: body mass index; SBP: systolic blood pressure; DBP: diastolic blood pressure; UAER: urinary albumin excretion rate; BUN: blood urea nitrogen; Cr: creatinine; TG: triglyceride; TC: total cholesterol. Statistical evaluation: *t*-test; ^∗^*P* < 0.05 versus the HC group.

**Table 2:** Hardy-Weinberg equilibrium test for the genotype distributions of IL-1*β* (-511C/T), IL-1*β* (+3953C/T), and IL-1Ra (+8006C/T) in the DN and HC groups.

Genotype	Group		*N*	Genotype (%)			*P*
				TT	TC	CC	
IL-1*β* (-511C/T)	DN	Measured	61	46 (75.4)	11 (18.0)	4 (6.6)	*χ* ^2^ = 1.612
		Theoretical		44 (71.0)	16 (25.8)	2 (3.2)	*P* = 0.45
	HC	Measured	48	22 (45.8)	19 (39.6)	7 (14.6)	*χ* ^2^ = 0.309
		Theoretical		21 (42.9)	22 (44.9)	6 (12.2)	*P* = 0.85
IL-1*β* (+3953C/T)	DN	Measured	61	16 (26.2)	24 (39.3)	21 (34.4)	*χ* ^2^ = 1.208
		Theoretical		13 (21.3)	30 (49.2)	18 (29.5)	*P* = 0.54
	HC	Measured	48	16 (33.3)	12 (25.0)	20 (41.7)	*χ* ^2^ = 6.443
		Theoretical		10 (20.8)	24 (50.0)	14 (29.2)	*P* = 0.05
IL-1Ra (+8006C/T)	DN	Measured	61	1 (1.6)	10 (16.4)	50 (82.0)	*χ* ^2^ = 0.304
		Theoretical		1 (1.6)	11 (17.7)	50 (80.6)	*P* = 1.00
	HC	Measured	48	1 (2.1)	8 (16.7)	39 (81.2)	*χ* ^2^ = 0.318
		Theoretical		1 (2.0)	9 (18.4)	39 (79.6)	*P* = 1.00

**Table 3:** The genotype and allele frequencies of IL-1*β* (-511C/T), IL-1*β* (+3953C/T), and IL-1Ra (+8006C/T) in the DN and HC groups (% of total).

Genotype or allele	DN	HC	*χ*2	*P*	OR (95% CI)
	(*N* = 61)	(*N* = 48)			
IL-1*β* (-511)					
TT	46 (75.4)	22 (45.8)	10.014	0.01^∗^	3.66 (0.968~13.827)
TC	11 (18.0)	19 (39.6)			1.01 (0.683~1.473)
CC	4 (6.6)	7 (14.6)			1.00
T	103 (84.4)	63 (65.6)	10.465	<0.01^∗∗^	2.84 (1.489~5.416)
C	19 (15.6)	33 (34.4)			1.00
IL-1*β* (+3953)					
TT	16 (26.2)	16 (33.3)	2.510	0.29	0.95 (0.378~2.401)
TC	24 (39.3)	12 (25.0)			1.91 (0.755~4.802)
CC	21 (34.4)	20 (41.7)			1.00
T	56 (45.9)	44 (45.8)	0.000	0.99	1.00 (0.586~1.715)
C	66 (54.1)	52 (54.2)			1.00
IL-1Ra (+8006)					
TT	1 (1.6)	1 (2.1)	0.318	1.00	0.78 (0.047~12.869)
TC	10 (16.4)	8 (16.7)			0.98 (0.352~2.703)
CC	50 (82.0)	39 (81.2)			1.00
T	12 (9.8)	10 (10.4)	0.020	0.89	0.94 (0.387~2.274)
C	110 (90.2)	86 (89.6)			1.00

∗ indicates *P* < 0.05; ∗∗ indicates *P* < 0.01.

## Data Availability

The data used to support the findings of this study are available from the corresponding author upon request.

## Supplementary Materials

Supplementary Materials Figure 1: IL-1*β* (-511C/T) genotyping plot. Figure 2: IL-1*β* (+3953C/T) genotyping plot. Figure 3: IL-1Ra (+8006C/T) genotyping plot.

## References

[1] Sagoo M. K., Gnudi L. (2020). Diabetic nephropathy: an overview. *Diabetic Nephropathy*.

[2] Rizvi S., Raza S. T., Mahdi F. (2014). Association of genetic variants with diabetic nephropathy. *World Journal of Diabetes*.

[3] Dinarello C. A. (2009). Immunological and inflammatory functions of the interleukin-1 family. *Annual Review of Immunology*.

[4] Garlanda C., Dinarello C. A., Mantovani A. (2013). The interleukin-1 family: back to the future. *Immunity*.

[5] McGeachy M. J., Bak-Jensen K. S., Chen Y. (2007). TGF-*β* and IL-6 drive the production of IL-17 and IL-10 by T cells and restrain T_H_-17 cell-mediated pathology. *Nature Immunology*.

[6] Dinarello C. A. (1996). Biologic basis for interleukin-1 in disease. *Blood*.

[7] Stenvinkel P. (2001). The role of inflammation in the anaemia of end-stage renal disease. *Nephrology Dialysis Transplantation*.

[8] Mantovani A., Locati M., Vecchi A., Sozzani S., Allavena P. (2001). Decoy receptors: a strategy to regulate inflammatory cytokines and chemokines. *Trends in Immunology*.

[9] Sims J. E., Smith D. E. (2010). The IL-1 family: regulators of immunity. *Nature Reviews Immunology*.

[10] Steinkasserer A., Spurr N. K., Cox S., Jeggo P., Sim R. B. (1992). The human IL-1 receptor antagonist gene (IL1RN) maps to chromosome 2q14-q21, in the region of the IL-1*α* and IL-1*β* loci. *Genomics*.

[11] Banerjee M., Saxena M. (2012). Interleukin-1 (IL-1) family of cytokines: role in type 2 diabetes. *Clinica Chimica Acta*.

[12] Maedler K., Sergeev P., Ris F. (2002). Glucose-induced beta cell production of IL-1beta contributes to glucotoxicity in human pancreatic islets. *The Journal of Clinical Investigation*.

[13] Lei Y., Devarapu S. K., Motrapu M. (2019). Interleukin-1*β* inhibition for chronic kidney disease in obese mice with type 2 diabetes. *Frontiers in Immunology*.

[14] Shahzad K., Bock F., Dong W. (2015). Nlrp3-inflammasome activation in non-myeloid-derived cells aggravates diabetic nephropathy. *Kidney International*.

[15] Salti T., Khazim K., Haddad R., Campisi-Pinto S., Bar-Sela G., Cohen I. (2020). Glucose induces IL-1*α*-dependent inflammation and extracellular matrix proteins expression and deposition in renal tubular epithelial cells in Diabetic kidney disease. *Frontiers in Immunology*.

[16] Khazim K., Azulay E. E., Kristal B., Cohen I. (2018). Interleukin 1 gene polymorphism and susceptibility to disease. *Immunological Reviews*.

[17] Lee S. H., Ihm C. G., Sohn S. D. (2004). Polymorphisms in interleukin-1*β* and Interleukin-1 receptor antagonist genes are associated with kidney failure in Korean patients with type 2 diabetes mellitus. *American Journal of Nephrology*.

[18] Alberti K. G., Zimmet P. Z., WHO Consultation (1998). Definition, diagnosis and classification of diabetes mellitus and its complications. Part 1: diagnosis and classification of diabetes mellitus provisional report of a WHO consultation. *Diabetic Medicine*.

[19] Hurme M., Santtila S. (1998). IL-1 receptor antagonist (IL-1Ra) plasma levels are co-ordinately regulated by both IL-1Ra and IL-1*β* genes. *European Journal of Immunology*.

[20] Dinarello C. A., Wolff S. M. (1993). The role of interleukin-1 in disease. *New England Journal of Medicine*.

[21] Tarlow J. K., Blakemore A. I. F., Lennard A. (1993). Polymorphism in human IL-1 receptor antagonist gene intron 2 is caused by variable numbers of an 86-bp tandem repeat. *Human Genetics*.

[22] Buraczynska M., Ksiazek K., Wacinski P., Zaluska W. (2019). Interleukin-1*β* gene (IL1B) polymorphism and risk of developing diabetic nephropathy. *Immunological Investigations*.

[23] Wetmore J. B., Hung A. M., Lovett D. H., Sen S., Quershy O., Johansen K. L. (2005). Interleukin-1 gene cluster polymorphisms predict risk of ESRD. *Kidney International*.

[24] Wang S., Dong J., Huang L. (2021). Cytokine polymorphisms and predisposition to diabetic nephropathy: a meta-analysis. *International Archives of Allergy and Immunology*.

[25] Niknami N., Omraninava M., Mirzaei N. (2018). Evaluation of the serum levels of IL-1 in type 2 diabetic patients with and without diabetic nephropathy. *Journal of Diabetes Mellitus*.

[26] Anders H.-J. (2016). Of inflammasomes and alarmins: IL-1*β* and IL-1*α* in kidney disease. *Journal of the American Society of Nephrology*.

[27] Donate-Correa J., Martín-Núñez E., Muros-de-Fuentes M., Mora-Fernández C., Navarro-González J. F. (2015). Inflammatory cytokines in diabetic nephropathy. *Journal of Diabetes Research*.

[28] Dinarello C. A., Simon A., Van Der Meer J. W. (2012). Treating inflammation by blocking interleukin-1 in a broad spectrum of diseases. *Nature Reviews Drug Discovery*.

[29] Stefanidis I., Kreuer K., Dardiotis E. (2014). Association between the interleukin-1*β* gene (IL1B) C-511T polymorphism and the risk of diabetic nephropathy in type 2 diabetes: a candidate-gene association study. *Dna & Cell Biology*.

[30] Iglesias Molli A. E., Bergonzi M. F., Spalvieri M. P., Linari M. A., Frechtel G. D., Cerrone G. E. (2020). Relationship between the IL-1*β* serum concentration, mRNA levels and rs16944 genotype in the hyperglycemic normalization of T2D patients. *Scientific Reports*.

