# Supplementary material for: Association of Interleukin-1 Beta and Interleukin-1 Receptor Antagonist Gene Polymorphisms and Plasma Levels with Diabetic Nephropathy
Source: Biomed Res Int. 2022 May 25;2022:9661823. doi: 10.1155/2022/9661823 (PMC9159863; doi:10.1155/2022/9661823)
Supplement: Supplementary Materials — Figure 1: IL-1β (-511C/T) genotyping plot. Figure 2: IL-1β (+3953C/T) genotyping plot. Figure 3: IL-1Ra (+8006C/T) genotyping plot. [file 9661823.f1.docx]

**Supplementary Materials:** **Association of interleukin-1 beta and interleukin-1 receptor antagonist gene polymorphisms and plasma levels with diabetic nephropathy**

**Xueling Liao, Yanan Xiao, Ulf Elbelt, Karsten H, Weylandt, Kanghui Li, Jie Deng, Ning Zeng, Chao Xue**


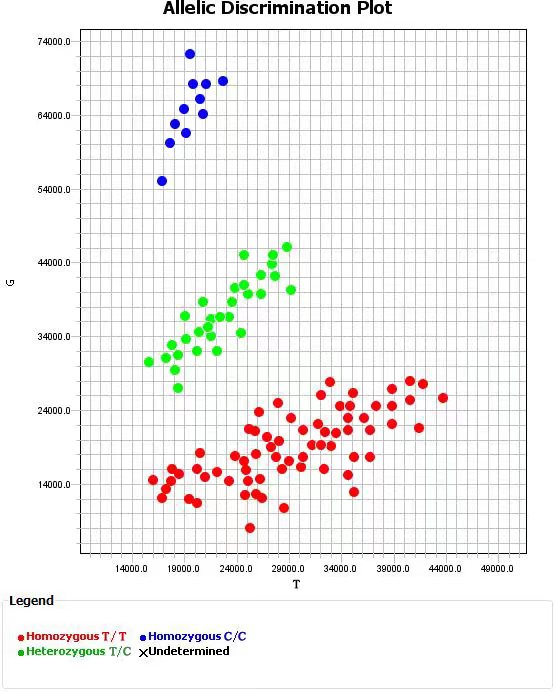


**Figure1.** IL-1β (-511C/T) genotyping plot


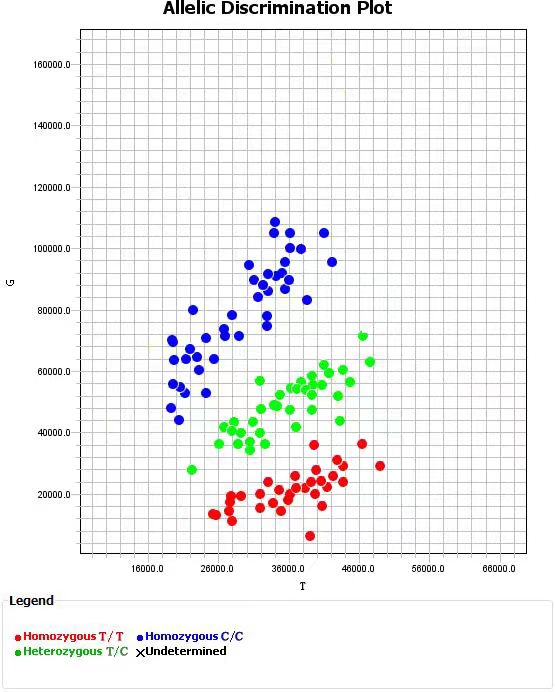


**Figure 2.** IL-1β (+3953C/T) genotyping plot


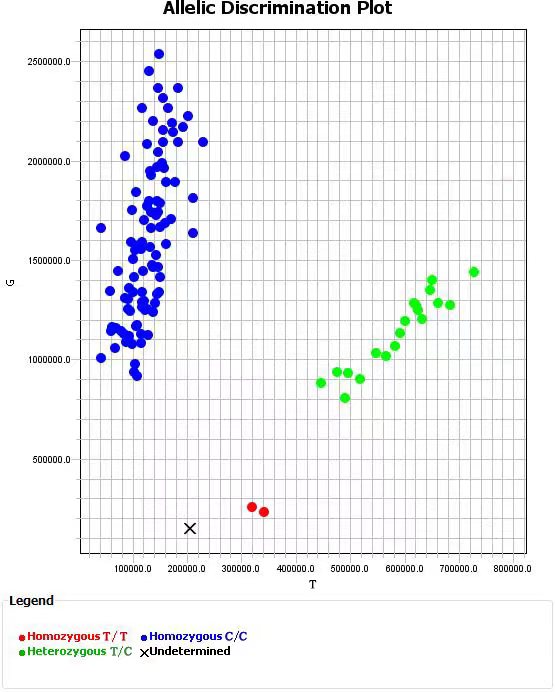


**Figure 3.** IL-1Ra (+8006C/T) genotyping plot
